# Functionalization of Electrospun Nanofiber for Bone Tissue Engineering

**DOI:** 10.3390/polym14142940

**Published:** 2022-07-20

**Authors:** Xuan Yan, Haiyan Yao, Jun Luo, Zhihua Li, Junchao Wei

**Affiliations:** 1 School of Stomatology, Nanchang University, Nanchang 330006, China; what118vicky@163.com (X.Y.); lizhihua@ncu.edu.cn (Z.L.); 2 School of Chemistry, Nanchang University, Nanchang 330031, China; ncuyaohaiyan@163.com; 3 Jiangxi Province Clinical Research Center for Oral Disease, Nanchang 330006, China; 4 Jiangxi Province Key Laboratory of Oral Biomedicine, Nanchang 330006, China

**Keywords:** electrospun nanofiber, bone-tissue engineering, functionalization, scaffold, bone regeneration

## Abstract

Bone-tissue engineering is an alternative treatment for bone defects with great potential in which scaffold is a critical factor to determine the effect of bone regeneration. Electrospun nanofibers are widely used as scaffolds in the biomedical field for their similarity with the structure of the extracellular matrix (ECM). Their unique characteristics are: larger surface areas, porosity and processability; these make them ideal candidates for bone-tissue engineering. This review briefly introduces bone-tissue engineering and summarizes the materials and methods for electrospining. More importantly, how to functionalize electrospun nanofibers to make them more conducive for bone regeneration is highlighted. Finally, the existing deficiencies of functionalized electrospun nanofibers for promoting osteogenesis are proposed. Such a summary can lay the foundation for the clinical practice of functionalized electrospun nanofibers.

## 1. Introduction

Bone is a hierarchical structure with outer cortical bone and inner cancellous bone [1,2,3]. Its unique and complex structure determines its vital functions: protection, support, movement and hematopoiesis [4]. The integrity and continuity of bone can be destroyed once it suffers from trauma, infection and tumor resection [5,6,7]. Generally, bone tissue has intrinsic regeneration ability [8,9], but this capacity has limitations. Clinical intervention is essential to help bone-defect healing.

The traditional intervention is bone graft, which can be classified into three categories according to donor bone source. The first is autologous bone graft, in which bone tissue obtained from the healthy bone of the patient is transplanted into the bone-defect area to promote bone healing. This has been the gold standard for bone repair [10,11,12] due to the excellent osteoconductivity and osteoinduction and low immunogenicity [13,14]. However, it destroys the integrity of normal bone tissue and brings pain to patients [15], which makes the scope of its implementation extremely narrow. The second is allogeneic bone graft, transplanting bone tissue from other people, especially the dead, into the bone defect site. This can remove damage to normal tissues, while it has a potential risk of immune rejection and disease transmission [16,17]. The third is xenogeneic bone graft, using mammals as a bone source; it is sufficient to meet high demand. Nonetheless, immune rejection and osteogenic infection restrict its comprehensive application [18,19]. Bone-tissue engineering (BTE) involves scaffolds, stem cells and growth factors to facilitate bone-defect repair, avoiding the drawbacks of bone graft. Currently, it is deemed as the ultimate solution to replace bone graft for repairing bone defects [20].

The scaffold is an essential component of BTE. Ideal BTE scaffolds are provided with mechanical and biochemical properties resembling the native bone tissues [21]. For example, good biocompatibility ensures dynamic scaffold–cell interaction. Suitable mechanical strength can withstand the external forces and support the new bone tissue [22]. Superb osteoconductivity and osteoinduction are conducive to new bone formation [23].

BTE scaffolds can be fabricated by diverse methods such as 3D printing [24], thermally induced phase separation, electrospinning and so on [25]. Among them, electrospinning has attracted extensive attention. Meanwhile, electrospun nanofibers have their distinctive advantages such as simulating the structure of ECM [26], which determines their crucial position in biomedical fields such as wound dressing [27], antibacterial [28], biosensor [29], drug delivery [30,31] and tissue engineering [32,33,34]. In addition, electrospun nanofibers can provide favorable environments for stem cells to perform significant physiological functions [35,36], which makes them suitable candidates for BTE scaffolds.

Electrospun nanofibers made from various materials have their inherent properties such as biocompatibility, biodegradability, and mechanical strength. In addition, electrospun nanofibers with diverse structures can be prepared via different spinning technologies, including blended electrospinning, multi-axial electrospinning, coaxial electrospinning and so on. However, the scaffolds manufactured by electrospinning are subject to bioactivity when applied in BTE. Therefore, a variety of approaches have been used to modify electrospun nanofibers to improve their properties for promoting bone-formation ability.

In this review, we first elaborate the methods, steps and constituents of BTE. We then generalize materials for electrospinning and their prospects in BTE. Next, we enumerate several electrospinning technologies and analyze their respective advantages. Subsequently, we focus on functionalization approaches optimizing the scaffold performance for bone regeneration. Finally, we propose future perspectives regarding functional electrospun nanofibers for BTE. It is expected that this review can provide reference for researchers and clinicians who are devoted to using electrospun nanofibers as scaffolds for BTE.

## 2. Basic Introduction about BTE

BTE is a novel and clinically viable therapy method for inducing bone regeneration [11]. Natural or artificial scaffolds containing stem cells related to osteogenesis are implanted into bone-defect areas to promote bone formation. The main steps are as follows: extract and isolate cells from a human body, culture cells to a specific number in vitro, fill these cells into the scaffolds, finally implant the scaffolds into bone defect areas. In general, the combination of scaffolds, growth factors and stem cells is intended to promote bone-defect healing.

Stem cell: A reliable stem cell source is indispensable in BTE for repairing bone defects. The most attractive stem cells for BTE are bone-marrow-derived mesenchymal stem cells and adipose-derived stem cells [37]. Stem cells should be characterized with the proliferation and differentiation potential and capacity for self-renewal so that highly differentiated functional cells can be obtained [38,39].

Growth factor: Growth factors exert an influence on inducing proliferation and differentiation of stem cells [40]. Bone morphogenetic proteins (BMPs), transforming growth factors-β (TGF-β), fibroblast growth factor (FGF) and vascular endothelial growth factor (VEGF) have great application potential in BTE [22]. Among them, BMP-2, a powerful osteoinductive factor, has the edge of inducing stem cells to differentiate into osteoblasts, gaining the favor of BTE.

Scaffold: scaffolds combined with stem cells and growth factors are implanted into organisms where they simulate the osteogenic microenvironment and provide accommodation for stem cells. The success of BTE relies on the performance of the scaffolds. The scaffolds should be degraded at the suitable time to provide growth space, bear pressure and promote osseointegration for restoring bone integrity.

Up to now, many different types of scaffolds, such as hydrogel [41], bioactive glass [42] and nanofiber [43], have application in BTE. Nanofibers are favored for their unconsolidated structure with large surface area. Furthermore, electrospun nanofibers have the advantages of simple preparation and controllable structure, so they are hopeful candidates for bone regeneration.

## 3. Materials Used for Electrospinning

Electrospun nanofibers can be obtained from natural materials, synthetic materials and composite materials. Natural materials interact with various types of cells without immune rejection. Synthetic materials cause immune response, but their mechanical property is outstanding. Making up for the shortcomings of natural materials and synthetic materials, the combination between materials is considered as a remediation.

### 3.1. Natural Materials

Natural materials are resourceful biomaterials with excellent biocompatibility. They are homologous with organisms and provide favorable biochemical signals to induce bone formation.

Chitosan (CS) originates from chitin-containing glucosamine and N-acetylglucosamine [43], which resemble glucosaminoglycans of bone in both structure and composition [44]. It has antibacterial and osteoconduction capacities, which are beneficial for BTE [45]. Hyaluronic acid is a natural polysaccharide, the principal element of ECM in mammalian connective tissue, which can provide a favorable osteogenic microenvironment [46]. It initiates many cellular signaling pathways and regulates cell activity and the release of biological factors to promote bone regeneration [47]. Collagen (COL) represents the organic component of bone tissue [4]. It is a feasible biomaterial for BTE due to its excellent osteoinduction [48]. Gelatin (Gel) is the product of partial hydrolysis of COL. It has a natural cell binding site like arginyl-glycyl-aspartic acid moieties (RGD peptide), and is instrumental in cell adhesion, proliferation, migration and differentiation [49,50]. Silk fibroin (SF) can simulate the anionic structure of noncollagenous proteins to provide precipitation sites for hydroxypatite (HAp) nanocrystals, which is beneficial to osteogenesis [51].

Natural materials can provide a three-dimensional (3D) structure for cell growth and bone-tissue formation [39]. However, their mechanical strength is poor.

### 3.2. Synthetic Materials

Synthetic materials, especially the degradable materials, are appreciated by BTE owing to their outstanding mechanical property. Moreover, these materials have the advantages of good malleability and easy processability, which can satisfy the requirements of various scaffolds. They are divided into two categories: organic polymer materials and inorganic nanomaterials.

Organic polymer materials are mainly used for matrix materials. Poly (ε-caprolactone) (PCL) with a semi-crystalline structure maintains excellent mechanical properties under the physiological conditions [52]. Moreover, PCL electrospun nanofibers enhanced the osteoblastic behavior of marrow stromal cells (MSCs) and accelerated calcium phosphate (CaP) mineralization [53]. Polylactic acid (PLA) has similar compressive strength to natural bone tissue [54] and appropriate biodegradation to provide growth space for stem cells. Poly (lactic-*co*-glycolic acid) (PLGA) is known for its excellent biocompatibility and biodegradability so its application in biomedical fields is approved by the Food and Drug Administration (FDA). Its flexible microstructure, controllable degradation rate and adjustable mechanical properties make it have the ability to satisfy different applications [55].

Inorganic nanomaterials promote osteointegration through interaction with the host, so they are used as additional materials. BTE especially has affinity for nanoceramics including bioactive HAp and bioresorbable tricalcium phosphate (TCP) [56]. HAp is the inorganic composition of bone matrix for increasing the tensile modulus of bone tissue through mineralization [13,57]. It exhibits strong affinity to bone tissue and is conducive to the dispersion and distribution of force for its larger surface area [58,59]. TCP has good compatibility with bone tissue [60]. TCP has a faster degradation rate than HAp so it produces more PO_4_^3−^ and Ca^2+^ to accelerate the mineralization of bone matrix and promote bone formation [61].

Although synthetic materials solve the problem of mechanical strength, their reduced bioactivity remains to be overcome. Therefore, the combination of materials is expected to achieve an ideal osteogenic effect.

### 3.3. Compound Materials

Compound materials are composed of two or more materials, which can compensate for their respective disadvantages to achieve good performance in BTE. The common methods to obtain compound materials include the combination of different kinds of polymers and the combination of polymers and nanoparticles. For example, electrospun PCL/poly (vinyl alcohol) (PVA) fibrous membranes containing metformin had a positive effect on human endometrial stem cells in promoting bone formation [62]. Gel-CS core-shell nanofibers enhanced the biological behavior of cells and improved the mineralization efficiency of HAp [63]. PCL/black phosphorus/COL nanofibers promoted the initial cell attachment, which is the key to cell adhesion. Meanwhile, the proliferation and osteogenic differentiation of preosteoblasts were enhanced [64]. Nanoparticles have large specific surface area, small size and good solubility, which endow them with the potential to improve the properties of scaffolds. For instance, carbonated hydroxyapatite nanoparticles were dispersed in the PVA/CS matrix, which can improve the biological activity [65]. Gel-PCL nanofibrous scaffolds precipitated with nanohydroxyapatite (nHAp) increased the adhesion and proliferation rate of osteoblasts [66]. SF scaffolds exhibited strong affinity to cell adhesion and proliferation but their application in BTE was limited by low mechanical strength. To overcome this issue, nHAp/SF elelctrospun composite nanofibers were fabricated for bone regeneration [67].

Compound materials can meet the requirements of BTE for mechanical strength and bioactivity, which makes up for the drawbacks of single materials, so they become ideal candidates for BTE scaffolds.

## 4. Methods of Electrospinning

**Figure 1:**
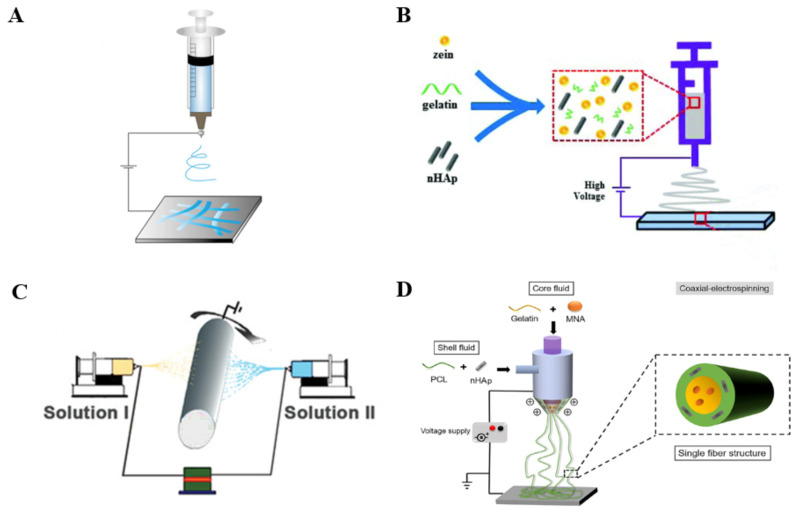
(**A**) Schematic of classical electrospinning setup. Reproduced with permission from [7]. Copyright Wiley 2021; (**B**) Schematic of blended electrospinning setup. Reproduced with permission from [73]. Copyright Remaking Singapore Committee 2019; (**C**) Schematic of multi-axial electrospinning setup. Reproduced with permission from [74]. Copyright Wiley 2020; (**D**) Schematic of coaxial electrospinning setup. Reproduced with permission from [76]. Copyright 2019, Elsevier.

Electrospinning is a technology with simple equipment to generate nanofibers from different materials [68]. Its typical device consists of a high-voltage power supply, a syringe pump, a spinneret and a collector (Figure 1A) [69]. In principle, it overcomes the surface tension of the polymer solution through repulsion produced by high voltage electricity [70]. Specifically, the solution is emitted from the spinneret and surface tension makes it turn into a droplet. The electrostatic repulsion generated by high voltage power supply makes the droplet deform and form a Taylor cone where a jet is spurted. After a series of movements, the jet is stretched into fibers with different diameters which accumulate on the collector with the evaporation of the solvent [52,71]. Many parameters affect this process such as solution concentration, voltage, distance between needle and collector, solution flow rate and so on [72]. Higher voltage, lower flow rate and longer distance between needle and collector can obtain finer fibers. The desired fiber diameter can be obtained by adjusting the parameters.

However, with classical electrospinning, it is difficult to meet the demands of applications. To endow electrospun nanofiber membranes with diverse functions, blended electrospinning, multi-axial electrospinning, coaxial electrospinning and some other electrospun technologies have been developed according to the trend.

### 4.1. Blended Electrospinning

Blended electrospinning is based on the co-dissolution of materials in specific medium. It makes materials intertwine with each other, improves the strength of the scaffold and endows the scaffold with more properties (Figure 1B). Zein, Gel and nHAp were homogeneously mixed for electrospinning to fabricate nanofiber membranes. The elctrospun zein/Gel/nHAp nanofiber membranes facilitated human periodontal ligament stem cells to attach, proliferate and differentiate towards osteoblasts [73].

### 4.2. Multi-Axial Electrospinning

Multi-axial electrospinning integrates the advantage of other materials and solves the problem of interaction between materials (Figure 1C). It can reasonably design the structure of nanofiber membranes for adaption to bone formation. A hierarchical Janus nanofiber membrane was prepared with multi-axial elelctrospinning for efficient bone formation. The random Gel fiber loaded with HAp is the inner face and the PCL solution modified with poly (methacryloxyethyltrimethyl ammonium chloride-co-2-Aminoethyl d2-methylacrylate hydrochloride) is the outer layer. The inner layer is favorable for the osteoblast adhesion, proliferation and osteogenic differentiation, and the outer layer prevented epithelial invasion and bacterial infection [74].

### 4.3. Coaxial Electrospinning

Coaxial electrospinning is characterized with a concentrically aligned dual nozzle: an inner core nozzle and an outer shell nozzle (Figure 1D) [75]. When two material solutions are injected simultaneously, nanofibers with shell-core structure can be formed [70]. Coaxial electrospinning can avoid the problem of phase separation of different components. Electrospun core-shell fibers can postpone drug delivery, so they are often used in BTE to control growth factors release. PCL and nHAp as shell along with Gel and metronidazole as core were electrospun into nanofiber membranes with enhanced osteogenesis and anti-infection [76].

### 4.4. Other Electrospun Technologies

In addition to the electrospinning technologies described above, melt electrospinning and melt electrowriting are widely employed to manufacture nanofibers [77]. Melt electrowriting is one of the most advanced technologies in constructing nanofibers in recent years, and is a promising additive manufacturing technology to produce 3D high-resolution scaffolds for bone regeneration [78,79,80]. Poly (L-lactic acid) (PLLA) was firstly fabricated by melt electrowriting into the scaffold with adjustable fiber diameter and pore size to obtain the optimal microenvironment for cell growth, which manifested enhanced bone formation [77].

It is universal that nanofibers prepared by the above-mentioned methods are applied in BTE. Chen et al. blended SF and CS for electrospinning to play a synergistic part in bone formation [81]. SF regulated stem cell proliferation and CS enhanced differentiation. The MTS assay exhibited an increasement of the human bone marrow mesenchymal stem cell proliferation. The Alizarin Red staining, alkaline phosphatase activity (ALP) and osteogenic marker gene expression showed the osteogenic differentiation of human bone marrow mesenchymal stem cells was enhanced. Picciani et al. developed PLA/PVA coaxial electrospun nanofibers as a sustained delivery platform for BMP-2 [82]. MTT tests showed core-shell PLA/PVA nanofibers enhanced cell proliferation rate and were not associated with BMP-2. Coaxial PLA/PVA nanofibers with BMP-2 performed high pre-osteoblast differentiation.

However, bone-tissue healing is a dynamic process which is variable over time. It is artificially divided into three stages: (1) inflammatory stage, (2) bone-formation stage, (3) bone-remodeling stage [13]. Shortening the inflammatory stage and prolonging the bone-formation stage can be artificially used to accelerate the healing of bone tissue. Their implementation is based on the scaffolds. Therefore, choosing appropriate scaffolds is the key to bone regeneration, while reprocessing and modifying the scaffolds can accelerate bone-tissue regeneration.

## 5. Functionalization of Electrospun Nanofiber for BTE

Electrospun nanofibers imitate the microenvironment of bone growth, so bone formation is definite. However, the osteogenesis ability of electrospun nanofibers is inferior due to the hydrophobicity and weak bioactivity of materials. In addition, bone regeneration is a chain reaction where many cells and multiple factors participate together. To accelerate bone regeneration, it is necessary to functionalize electrospun fibers to improve their properties. Surface functionalization is an effective post-modification method including physical modification and chemical modification.

### 5.1. Physical Modification

Physical modification is an available method without special equipment and does not produce by-products. It attempts to introduce functional substances on the electrospun nanofiber membrane surface to improve hydrophilicity and bioactivity. Physical surface deposition and layer-by-layer (LbL) self-assembly are two main approaches of physical modification.

#### 5.1.1. Physical Surface Deposition

Physical surface deposition is a simple and feasible approach driven by electrostatic interaction, hydrogen bond and van der Waals force [83]. The modified nanofiber membrane can be mainly obtained by immersing the nanofiber membrane in the solution. The modified nanofiber membrane can guide the adhesion, proliferation and differentiation of stem cells and further accelerate bone formation.

Many substances are deposited on the surface of electrospun nanofibers by physical force, which can improve the osteogenic ability and promote the mineralization of the bone matrix. For example, electrospun PLA nanofibers were immersed in CS aqueous solution to form cationic CS coating, which promoted nucleation and growth of calcium phosphate [84]. Many HAp crystals occurred on the surface of CS-coated PLA nanofibers. The calcium/phosphorus ratio increased extremely from 1.35 to over 1.60 in the CS-coated groups. The intracellular ALP level increased when the PLA nanofibers were coated with CS. These results suggest that CS-coated PLA nanofibers promoted bone-tissue biomineralization.

**Figure 2:**
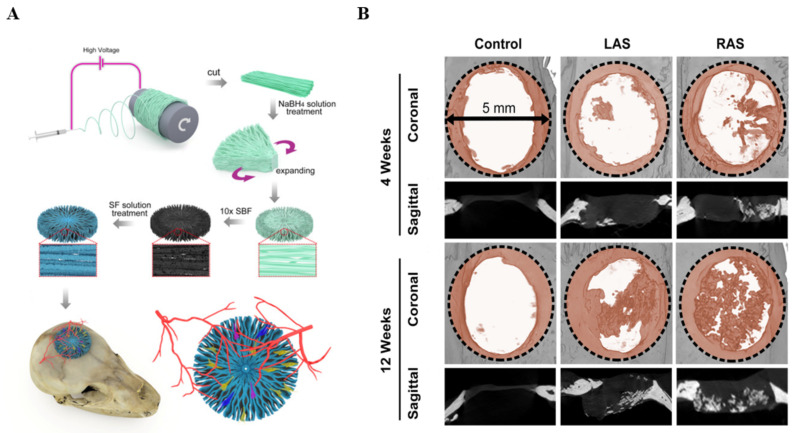
(**A**) Fabrication and application of the nanofiber scaffolds with SF and HAp coating; (**B**) Micro-CT images of the defect areas for 4 and 12 weeks. Reproduced with permission from [86]. Copyright 2021, Elsevier.

Calcium phosphate (CaP) can be deposited on electrospun nanofibers to directly accelerate bone mineralization. Mavis et al. immersed PCL electrospun nanofiber mats in modified 10SBF solutions to form CaP nanocoating on the nanofiber surfaces [85]. MTT assay showed MC3T3-E1 cells rapidly attached and proliferated on the PCL-CaP composite nanofiber mats during the first 12 days. The intracellular ALP activity of MC3T3-E1 cells presented higher expression in PCL nanofibers coated with CaP. A great deal of osteocalcin was detected on PCL nanofibers coated with CaP. Cai et al. used SF and HAp as coatings to obtain radial 3D PCL nanofibers with mineralization capacity to repair bone defects [86]. PCL nanofiber scaffolds were hydrolyzed to achieve the surface which had affluent carboxylic acid. Then, HAp and SF were successively deposited on the scaffold surface (Figure 2A). Micro-CT of critical cranial defects in a rat model was applied to assess the bone regeneration effect. The results showed that the modified scaffolds had more bone formation and greater bone closure (Figure 2B).

Although physical surface deposition provides a significant improvement in the osteogenic properties of electrospun nanofibers, bonding instability between coating and electrospun nanofibers restricts its extensive application.

#### 5.1.2. Layer-by-Layer (LbL) Self-Assembly

LbL self-assembly precipitates substrates on the surface of electrospun nanofibers through electrostatic interaction, hydrogen bond and conjugate interaction to form multi-layer coatings [87]. Owing to its wide application, LbL self-assembly technology has developed rapidly, including immersive assembly, spinning, spraying, immobilization and so forth [88]. Immersive assembly is the main application form. Its universality for all substrates has attracted considerable attention in biomedical fields [83].

**Figure 3:**
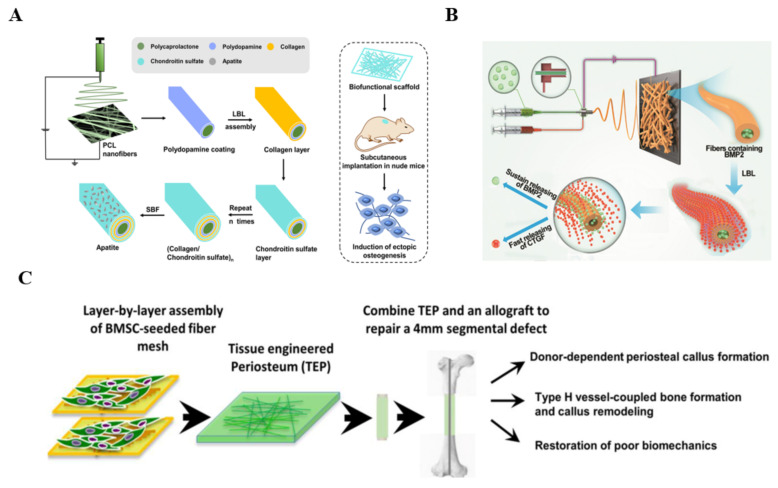
(**A**) Schematic of fabrication of the artificial scaffold with biofunction through LbL self-assembly. Reproduced with permission from [87]. Copyright 2021, Elsevier; (**B**) Schematic of preparation of the SF/PCL/PVA coaxial fibers with BMP-2 and CTGF via LbL for BTE. Reproduced with permission from [89]. Copyright American Chemical Society 2019; (**C**) Schemes of fabricating tissue-engineered periosteum (TEP) and application for bone defects. Reproduced with permission from [90]. Copyright 2018, Elsevier.

LbL self-assembly is intended to fabricate the multilayer structure to accelerate the process of bone formation. For example, Lvov et al. deposited type-I COL and chondroitin sulfate via LbL self-assembly on electrospun PCL fibers modified by polydopamine and then induced apatite precipitation in situ to design an artificial scaffold [87] (Figure 3A). LbL-modified PCL fibers induced a faster mineralization because COL could regulate mineralization. MC3T3-E1 cells incubated on the modified fibers showed higher cell adhesion and excellent proliferation ability. ALP activity, type I COL secretion and calcium deposition results indicated that the modified PCL fibers had the better ability to induce osteogenic differentiation, which was consistent with in vivo ectopic osteogenesis.

LbL-modified electrospun nanofiber membranes are a useful drug-delivery carrier to controllably release growth factors. SF/PCL/PVA nanofiber mats were fabricated into a core-shell structure using coaxial electrospinning. BMP-2 was incorporated into a PVA solution as the core and connective tissue growth factor (CTGF) was immobilized on the nanofiber mats via LbL self-assembly technology, which achieved the slow release of BMP-2 and the burst release of CTGF to promote vessel and bone formation [89] (Figure 3B). ALP and type I COL of MSCs were highly expressed on nanofibers containing BMP-2, indicating that BMP-2 had the ability to induce osteogenesis. VEGF had a higher expression on LbL-modified nanofibrous mats, indicating CTGF indirectly promoted angiogenesis. Histopathological results showed that co-delivery of BMP-2 and CTGF based on LbL self-assembly promoted microvessels and bone formation.

To optimize the quality of bone healing, Zhang et al. combined PCL, COL and nHAp to prepare a biomimetic tissue-engineered periosteum (TEP) seeded with bone mesenchymal stem cells (BMSCs) through layer-by-layer bottom-up strategy for restoring the structure and function of bone tissue [90] (Figure 3C). The bone defect mouse model healing results revealed that TEP restored periosteal bone formation at the defect site.

The effect of LBL-modified electrospun nanofibers on promoting osteogenesis is apparent. However, the fact that it is a time-consuming and laborious technology must be admitted.

### 5.2. Chemical Modification

Physical modification is prone to be affected by the environment and their properties may be changed easily, so it is difficult to achieve a stable osteogenic effect. Chemical modification is employed to overcome this issue because it can form strong forces on nanofibers to resist external forces. The commonly used chemical modification approaches include plasma treatment, grafting and crosslinking.

#### 5.2.1. Plasma Treatment

Plasma treatment is a special type of surface functionalization method to improve hydrophilicity by introducing polar groups [91]. A variety of gases such as oxygen, nitrogen, ammonia, argon, hydrogen and carbon dioxide are introduced in the process of plasma treatment to optimize the performance of scaffolds [92]. Gas species and treatment time have an important influence on the effect of plasma treatment [93]. The largest number of ideal functional groups can be obtained by reasonably selecting plasma resources and controlling time.

Plasma treatment indirectly enhances cell behavior by improving the hydrophilicity of electrospun nanofibers to promote bone formation. For example, electrospun PCL nanofiber meshes were treated with plasma to observe the effects on cell adhesion and proliferation [94]. Water contact angle measurement results analysis shows that plasma treatment considerably increased the wettability of electrospun nanofibers. Osteoblast-like cells presented enhanced cell adhesion and faster proliferation. Habibovic et al. treated electrospun PolyActive meshes, a family of block copolymers of poly (ethylene oxide terephthalate) and poly (butylene terephthalate) with tunable properties, with oxygen plasma to assess whether the proliferation and osteogenic differentiation of human bone marrow mesenchymal stem cells would be affected [95]. The improvement in hydrophilicity was apparent and the expression of ALP on plasma-treatment meshes was higher than that on untreated meshes. Plasma-treated surfaces had a positive effect on the expression of osteogenic genes, osteonectin and bone sialoprotein after culture for 7 days and on the activity of ALP after culture for 21 days. Tabaei et al. incorporated coral into electrospun CS/polyethylene oxide nanofibers and then functionalized them with plasma, which enhanced their wettability and osteogenic performance [96]. Compared with untreated nanofibers, the cell adhesion and proliferation on plasma-treated nanofibers were significantly improved and the amount of calcium phosphate deposition increased. These results confirmed that the plasma-treated electrospun nanofibers had excellent osteogenic properties.

**Figure 4:**
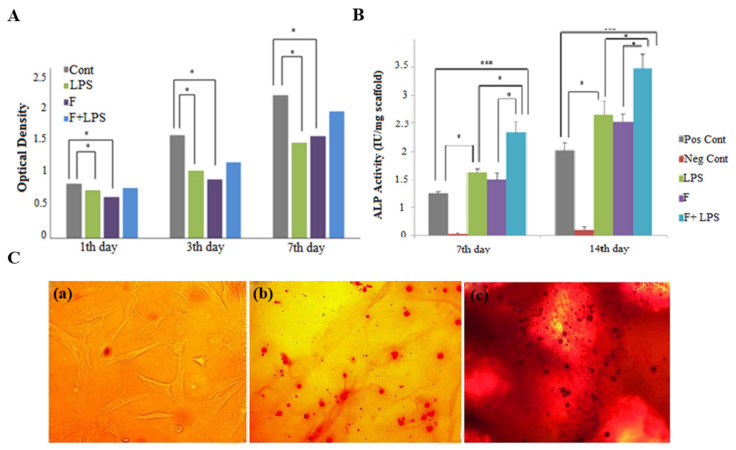
(**A**) Proliferation of MSCs on different scaffolds cultured for 7 days; (**B**) Alkaline phosphatase (ALP) activity in 2 weeks; (**C**) Alizarin red staining of MSCs on different scaffolds cultured in osteogenic culture medium for 14 days, * *p* ≤ 0.05, *** *p* ≤ 0.001. Reproduced with permission from [97]. Copyright Wiley 2018.

In addition, plasma treatment can combine with other methods to further promote osteogenesis. For instance, PLLA electrospun nanofibers were treated with O_2_ plasma and preconditioned with lipopolysaccharide to obtain increased hydrophilicity and the potential to induce cell attachment, growth and differentiation [97]. MTT assay results confirmed the ability of modified PLLA nanofibers to enhance MSC proliferation (Figure 4A). ALP activity of modified PLLA nanofibers was the highest (Figure 4B). Alizarin red staining of MSCs detected a great amount of calcium deposition on modified PLLA nanofibers (Figure 4C). Albayrak et al. modified Poly(3-hydroxybutyrate-co-3-hydroxyvalerate) (PHBV) through plasma treatment to improve hydrophilicity and immobilized SF to provide recognition sites for cells to promote bone regeneration [98]. The modified PHBV nanofiber mat surface presented a high coverage with calcium and phosphorus precipitations. MTT assay results revealed cell adhesion and proliferation were considerable on the modified PHBV nanofiber mat. ALP activity of the modified PHBV nanofiber mat was higher than the PHBV nanofiber mat, which meant better osteogenic differentiation.

Plasma treatment is an environment-friendly technology without adding chemical reagent. However, the improved hydrophilicity of electrospun nanofibers through plasma treatment is not very stable; after a few hours, the effect of plasma treatment may already vanish; therefore, stable and long lasting modification methods are imperative.

#### 5.2.2. Grafting

Grafting immobilizes specific substances on the surface of polymers through chemical bonds, based on functional groups such as carboxyl, hydroxyl, anhydride and so on. Chemical grafting can be obtained by two methods. The first type is radical initiated surface grafting, such as atom transfer radical polymerization (ATRP). The second is copolymerization grafting which simply introduces specific substances on the surface of nanofiber membranes by chemical linkage [92].

In the free radical initiated surface grafting process, the nanofiber surfaces are locally activated by the free radicals generated by the initiator and combine with specific substances to form graft polymers. Grafting polymers with improved hydrophilicity have attracted enormous attention in various areas, especially BTE. Apohan et al. fabricated electrospun chitosan fibers grafted with poly (2-methacryloyloxyethyl phosphorylcholine) (MPC) by ATRP in order to enhance the biological performance of osteosarcoma cells [99]. SEM images clearly showed the cell adhesion and spread on the chitosan fibers grafted with poly (MPC). MTT results demonstrated chitosan fibers grafted with poly (MPC) were suitable for cell attachment. Osteosarcoma cells with some characteristics of osteoblast proliferation on the chitosan fibers grafted with poly (MPC) were obvious due to their high sensitivity to phosphorylcholine groups. These results showed that poly (MPC)-grafted chitosan fibers were conducive to osteogenesis.

**Figure 5:**
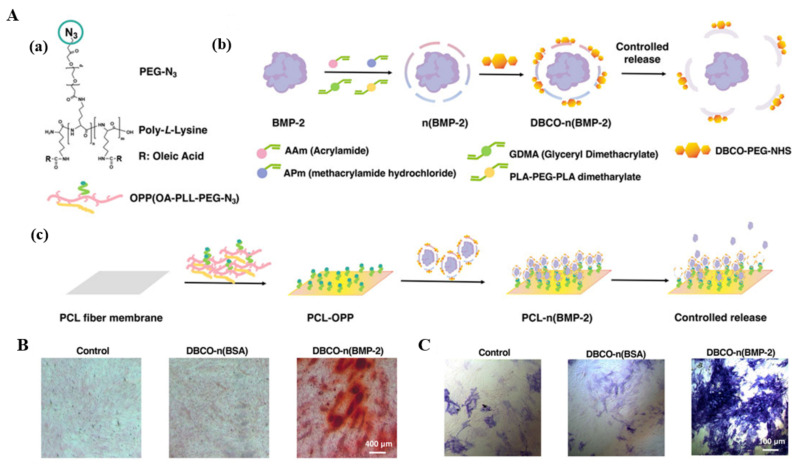
(**A**) (a) Schematic diagram of chemical structure of grafted polymer OA-PLL-PEG-N3 graft polymer; (b) Schematic of synthesis process about dibenzocyclooctyne-modified BMP-2 nanocapsules; (c) Schematic of surface functionalization on the surface of PCL scaffold for controlling the release of growth factor; (**B**) Illustration of Alizarin Red Staining results of BMSCs for 10 days; (**C**) Illustration of the ALP staining results of BMSCs. Reproduced with permission from [101]. Copyright Frontiers 2022.

Copolymerization grafting is a mild, simple and efficient approach. For example, a PCL nanofiber was modified by acrylic acid plasma to generate O-based functional groups to enhance cell–scaffold interaction. Then, ethylene diamine was grafted on the carboxylic acid groups of PCL nanofiber to enhance the behavior of bone marrow mesenchymal stem cell [100]. Bone marrow mesenchymal stem cells on the modified PCL nanofiber spread homogenously and completely covered the surface. Cells proliferated well and distributed layer by layer on the modified PCL nanofibers. Yu et al. prepared electrospun PCL nanofibers grafted with an azide-terminated amphiphilic graft polymer to overcome their native hydrophobicity. Then dibenzocyclooctyne-modified growth factor nanocapsules were employed to further functionalize grafted PCL nanofibers through click chemistry, achieving the controllable release of BMP-2 (Figure 5A) [101]. The water contact angle of grafted PCL nanofibers decreased from 122.65° to 31.45°, indicating that hydrophilicity was greatly improved. Alizarin red staining showed the strongest redness intensity, suggesting better ossification (Figure 5B). The ALP activity was the highest in functionalized PCL nanofibers (Figure 5C).

Grafted polymers can be stably bonded with electrospun nanofibers and improve their properties or endow them with additional function. Obviously, grafting has a great prospect in improving osteogenic ability.

#### 5.2.3. Crosslinking

Crosslinking refers to coupling two or more molecules with crosslinking agents to form a whole for maintaining steadiness. 1-(3-Dimethylaminopropyl)-3-ethylcarbodiimide hydrochloride (EDC)/N-hydroxysulfo-succinimide sodium salt (NHS) is the widely used crosslinking agent in electrospun nanofibers. In addition, crosslinking is also used to bind other molecules related to osteogenesis in electrospun nanofibers.

**Figure 6:**
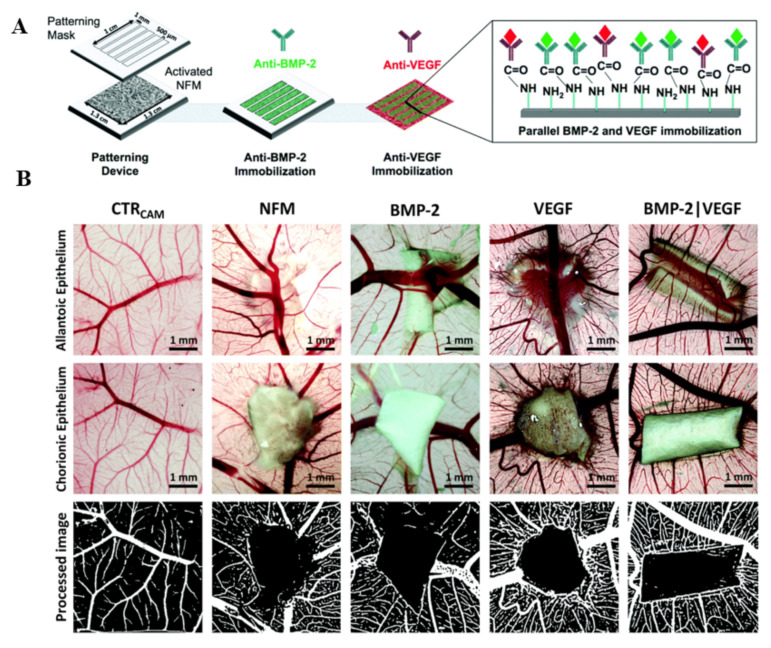
(**A**) Schematic of activated electrospun PCL nanofibers immobilized with anti-BMP-2 and anti-VEGF; (**B**) Schematic of vascularization in bone defect area. Reproduced with permission from [103]. Copyright Remaking Singapore Committee.

For example, ascorbic acid, β-glycerophosphate disodium salt hydrate, HAp and Gel were blended for electrospinning. Then the scaffold was crosslinked by NHS and EDC to form an entirety [102]. Gel-HAp electrospun nanofiber scaffolds modified by ascorbic acid and β-glycerophosphate disodium salt hydrate created an osteoconductive and osteoinductive microenvironment for bone regeneration. The scaffolds showed higher Runx2, Sp7, Alp and Col1 expression. Animal assay demonstrated that 94% of new bone tissue formed in the modified scaffold at 6 weeks post-surgery, which was very much higher than other scaffolds. Neves et al. immobilized anti-BMP-2 and anti-VEGF on an electrospun PCL nanomembrane via a coupling agent EDC/NHS (Figure 6A). Then, BMP-2 and VEGF were bound to anti-BMP-2 and anti-VEGF on the electrospun PCL membrane, respectively [103]. Human bone marrow-derived mesenchymal stem cells exhibited higher ALP activity and expressed endothelial-related genes on a PCL membrane containing BMP-2 and VEGF. The chick chorioallantoic membrane assay results showed the sprouting of mature vasculature on the PCL membrane containing BMP-2 and VEGF (Figure 6B). They confirmed that vascularized BTE could be obtained by crosslinking.

Crosslinking takes advantage of chemical force to anchor functional molecules on electrospun nanofibers. For instance, a biomimetic Janus chitin nanofiber membrane was envisaged for guiding bone regeneration. MPC was copolymerized with trimethoxyloypropyl methacrylate through free radical copolymerization to fabricate crosslinking sites which would be anchored with amino and hydroxyl groups of chitins to inhibit the growth of soft tissue. Chitin nanofibers promoted new bone formation [104]. In vitro cell test results demonstrated nanofiber membrane prevented excessive fibroblast infiltration in the bone-defect area. ALP activity on chitin nanofibers showed an increasing trend over time and was higher than the control group. Zhang et al. fabricated the PCL/Gel hybrid nanofibrous membrane and then crosslinked with genipin to enhance osteogenesis capability [105]. The CCK-8 results indicated the nanofiber membrane had good biocompatibility. A large amount of calcium deposition was covered on hybrid membranes, indicating that the scaffold was favorable for osteogenesis. Yang et al. developed electrospun Ag-CaP/CS nanofiber membranes crosslinked with vanillin to guide bone regeneration [106]. Bone mesenchymal stem cells showed good attachment and growth as well as improved proliferation on the membrane surface, suggesting modified nanofiber membranes provided an osteoinductive environment for bone formation.

Crosslinking agents enhance the water stability [92] and osteogenic performance of electrospun nanofibers without destroying other properties. Therefore, crosslinking is a suitable approach for tailoring the osteogenic capacity of electrospun nanofibers.

## 6. Conclusions

Electrospun nanofibers have attracted extensive attention in biomedical field due to their porosity and large specific surface area, whereas their application in BTE is confined owing to lack of bioactivity. Functionalization approaches have occurred as a flexible strategy to improve their properties such as hydrophilicity, stability and biological behavior. Several studies have confirmed their effect, parts of which were discussed in this review.

In general, electrospun nanofiber membranes can be modified by physical or chemical approaches. Physical approaches are characterized by simple operation and mild conditions, but their interactions are so unstable that they cannot achieve long-term osteogenic effect. Chemical approaches including plasma treatment, grafting and crosslinking have a good prospect in BTE. Chemical approaches to modify electrospun nanofibers are superior to physical approaches in tissue engineering. Because the functional groups or the molecules are bound to the electrospun nanofibers via covalent bond, they are too stable to leach out from the surface of modified nanofibers. Even though every effort has been made to improve cell–scaffold interactions and great progress has been made, there are still deficiencies concerning functionalized electrospun nanofibers for BTE.

Early vascular formation and the homeostasis of bone formation and bone resorption are critical for bone-tissue repair. Functionalized electrospun nanofibers do not emphasize the affinity for vascular endothelial cells, so they are not conducive to forming vascularized bone. The receptors of vascular endothelial cells should be embedded on the surface of electrospun nanofiber membranes to recognize vascular endothelial cells and facilitate neovascularization to provide nutrition for the new bone tissue. Better adjusting the balance between osteogenesis and osteoclasty, molecules targeted at inhibiting osteoclast behavior should be integrated with functionalized electrospun nanofibers.

In summary, functionalization has greatly improved the biochemical properties of electrospun nanofibers, making them regain a good reputation in BTE. However, functional electrospun nanofibers applied in clinical therapy have a long way to go. For example, their safety has not been confirmed and the degradation mechanism is not clear. It is certain that one day they will be used in clinical practice owing to their excellent performance.

## Data Availability

The data presented in this study are available on request from the corresponding author.
